# Does Corpus Luteum Doppler Have a Role in Prognostic Prediction for Outcome with Threatened Abortion?

**DOI:** 10.3390/jcm14051419

**Published:** 2025-02-20

**Authors:** Gizem Aktemur, Betül Tokgöz Çakır, Sadun Sucu, Gülşan Karabay, Zeynep Şeyhanlı, Nazan Vanlı Tonyalı, Elif Gülşah Diktaş, Mehmet Ünsal, Salim Erkaya

**Affiliations:** 1 Department of Obstetrics and Gynecology, Division of Perinatology, Ankara Etlik City Hospital, 06170 Ankara, Turkey; btltkgz@gmail.com (B.T.Ç.); drssucu@gmail.com (S.S.); drgulsankarabay@gmail.com (G.K.); drzeynepseyhanli@gmail.com (Z.Ş.); nazanvanli@gmail.com (N.V.T.); 2 Femart Clinic, 06510 Ankara, Turkey; elifgulsahdiktas@gmail.com; 3 Department of Obstetrics and Gynecology, Ankara Bilkent City Hospital, 06800 Ankara, Turkey; munsal174@hotmail.com (M.Ü.); salimerkaya59@gmail.com (S.E.)

**Keywords:** corpus luteum Doppler, threatened abortion, subchorionic hemorrhage, pregnancy outcomes, first-trimester prognosis

## Abstract

**Background/Objectives**: This study evaluated the prognostic value of corpus luteum Doppler findings in predicting pregnancy outcomes in patients with threatened abortion, with or without subchorionic hemorrhage. **Methods:** A prospective cohort study included 180 pregnant women diagnosed with threatened abortion in the first trimester—120 without subchorionic hemorrhage and 60 with subchorionic hemorrhage. Corpus luteum Doppler parameters, including systole/diastole ratio, Resistance Index, and Pulsatility Index, were measured via transvaginal ultrasonography. Pregnancy outcomes were categorized as first-trimester abortion or continuation into the second trimester. **Results:** Corpus luteum Doppler parameters were significantly higher in patients with first-trimester abortion compared to those with ongoing pregnancies (*p* < 0.001). Among patients with subchorionic hemorrhage, those who experienced abortion also showed higher Doppler values (*p* < 0.001). ROC analysis indicated that corpus luteum Doppler parameters effectively predicted first-trimester abortion, with a systole/diastole ratio > 2.87 achieving 77% sensitivity and 75% specificity (AUC = 0.767, *p* < 0.001). **Conclusions:** Corpus luteum Doppler is a valuable, non-invasive tool for predicting pregnancy outcomes in threatened abortion, particularly with subchorionic hemorrhage. Increased resistance in Doppler parameters may indicate luteal insufficiency and reduced progesterone levels. Larger studies are needed to confirm these findings and explore underlying mechanisms.

## 1. Introduction

Vaginal bleeding and a blocked cervical canal during the first trimester of pregnancy are classified as a threatening miscarriage [1]. A threatening miscarriage is an adverse obstetric condition that impacts one-fifth of all pregnancies. This condition is generally not regarded as a serious obstetric issue and lacks significant repercussions. It can increase the emotional burden on the mother and is one of the main reasons why pregnant women visit the hospital [1,2]. It has also been said to be associated with adverse maternal and neonatal outcomes in some cases [3]. Consequently, numerous biochemical, ultrasonographic, and clinical markers were evaluated to forecast the pregnancy outcome [2,4].

Subchorionic and subamniotic hemorrhages, classified as intrauterine hemorrhages, frequently arise in patients diagnosed with a threatening miscarriage and are identified via ultrasonography. The rate of vaginal bleeding in patients is between 4% and 22% [5]. These hematomas are usually visible on ultrasound as hypoechoic or echoless areas. The exact cause is unknown, but they are thought to be caused by a partial detachment of the chorionic membranes from the uterine wall [6]. The clinical relevance of these hematomas remains unclear; nevertheless, a meta-analysis indicates that the presence of a subchorionic hematoma doubles the chance of pregnancy loss during both early and late trimesters [7,8].

Vaginal Doppler ultrasonography is a straightforward, readily available, and cost-effective technique that can be used throughout the first trimester of gestation. Blood flow to the uterine arteries and corpus luteum may be quantified in 100% and 75% of pregnant women, respectively [9]. Abnormal Doppler results in the uteroplacental circulation during the early trimester correlate with adverse pregnancy outcomes [10]. Nevertheless, certain investigations have indicated that there is no distinction in Doppler findings between women experiencing a threatening miscarriage and those not undergoing miscarriage [11,12].

Progesterone is essential for proper implantation and the maintenance of pregnancy during the first trimester. The primary known function of the corpus luteum is to supply progesterone until placental circulation is established [13]. Transvaginal ultrasound is a commonly utilized technique for evaluating the corpus luteum (CL) and its vascularity during early pregnancy, as corpus luteum blood flow is crucial for the development of the CL and the preservation of luteal function [14]. Research indicates that the capacity of the corpus luteum to synthesize progesterone is closely correlated with its vascular perfusion. Increased blood flow to the corpus luteum correlates with heightened progesterone production. Prior research indicates that diminished corpus luteum blood flow may signify compromised luteal function, resulting in inadequate progesterone synthesis, which could subsequently lead to pregnancy loss. The vascularization of the CL is directly correlated with its progesterone synthesis capacity; thus, evaluating CL Doppler indices may yield indirect information regarding luteal function and its influence on pregnancy viability. This study seeks to elucidate the potential significance of CL blood flow in early pregnancy difficulties by the examination of this relationship [15]. In addition to ultrasound studies, many biomarkers have been studied to predict pregnancy outcomes in pregnant women presenting for threatened miscarriage. In a meta-analysis of biomarkers studied to determine pregnancy outcomes in these pregnant women, it was found that there are conflicting results that require further review and investigation [16].

The primary objective of this study was to determine the prognostic value of CL Doppler measurements in predicting pregnancy outcomes in threatened abortion cases. In this context, CL Doppler parameters (S/D ratio, RI, and PI) were compared between patients who had an abortion in the first trimester of pregnancy and those who continued their pregnancy, and the relationship between the presence and size of subchorionic hematoma and these parameters was evaluated. The secondary objective was to examine the threshold values determined using ROC analysis in order to determine the sensitivity and specificity rates of CL Doppler measurements, to investigate their effects on pregnancy prognosis, and to analyze survival rates according to gestational age. In this context, the primary endpoints in the study were pregnancy loss rate (first-trimester miscarriage and continuing pregnancy), CL Doppler parameters (S/D ratio, RI and, PI) and presence and size of subchorionic hematoma. Secondary endpoints included survival rate according to gestational age, sensitivity and specificity rates of threshold values determined by ROC curve analysis, and distribution of Doppler measurements between groups experiencing and continuing pregnancy loss. These specific endpoints were used to demonstrate the prognostic value of CL Doppler measurements and aim to contribute to the management of threatened miscarriage.

## 2. Materials and Methods

### 2.1. Study Design

This prospective observational cohort study was conducted with 180 patients who were diagnosed with threatened abortion between 2020 and 2021 at Ankara Etlik Zübeyde Hanım Gynecology and Obstetrics Training and Research Hospital. Informed consent was obtained from all women after a full explanation of the objectives of the study. The research protocol was approved by the Ethics Committee of Etlik Zübeyde Hanım Gynecology and Obstetrics Training and Research Hospital (protocol number: 900-57706799).

### 2.2. Study Participants

Patients admitted to the hospital with a diagnosis of threatening abortion, in the first trimester of pregnancy, exhibiting a closed cervical canal, vaginal bleeding, and/or active pelvic pain were evaluated. Patients exhibiting a crown-rump length (CRL) of 6–12 weeks of fetal cardiac activity, with or without a subchorionic hemorrhage, were selected. Patients with comorbidities, gynecological disorders including fibroids, adenomyosis, ovarian cysts, unexplained infertility, those who conceived via assisted reproductive technologies, and individuals diagnosed with recurrent pregnancy loss were excluded from the study. Also, patients were excluded from the study if their pregnancy follow-up was not conducted at our institution or if their information was inaccessible after the 20th week of gestation. The study comprised 120 pregnant women devoid of subchorionic hemorrhage and 60 pregnant women with subchorionic or subamniotic hemorrhage.

### 2.3. Ultrasonography and Doppler Study Protocol

All Doppler ultrasonography measurements were performed by a single experienced sonographer using a Samsung HS70A transvaginal ultrasound device (North Charleston, SC, USA). The measurements were obtained using a 4–9 MHz transvaginal probe, with all pregnant women evaluated in the lithotomy position with an empty bladder.

#### 2.3.1. Corpus Luteum (CL) Localization and Identification

The adnexal region was thoroughly examined to determine the location of the corpus luteum (CL). The CL was identified as a hypoechoic ring-like structure, and its surrounding vascular architecture was visualized using color Doppler imaging. In all included pregnancies, it was ensured that only a unilateral CL was present.

#### 2.3.2. Doppler Measurement Procedure

Color Doppler scanning was used to map the vascular structures surrounding the CL, and the region with the most prominent blood flow was selected. Power Doppler mode was applied to enhance the assessment of vascularization. In spectral Doppler analysis, three consecutive waveform recordings were obtained and analyzed. The following Doppler indices were measured:

S/D ratio (Systolic/Diastolic ratio)
RI (Resistance Index)
PI (Pulsatility Index)

#### 2.3.3. Measurement Standards and Bias Reduction Strategies

To minimize angular measurement errors, the Doppler insonation angle was maintained between 0 and 60 degrees. Partial volume settings were optimized (minimum width of 2 mm) to ensure that only intra-vessel flow was measured. Doppler settings were adjusted by keeping the wall filter at a low level and maintaining an optimal pulse repetition frequency (PRF).

All ultrasonography and Doppler assessments were conducted by a singular, competent sonographer specifically trained for the project, thereby minimizing observer bias. Secondly, participants were selected according to well-defined inclusion and exclusion criteria; patients with comorbidities or other gynecological problems were excluded from the study. All patients included in the study were followed until the end of pregnancy, and patients who fell outside the criteria were excluded during this time. Thus, selection bias and evaluation bias were reduced. To assess the intra-observer reliability of Doppler measurements, a subset of 10% of the study participants was randomly selected for repeated measurements. ICC was previously studied by Koo T.K. et al. To assess the intraobserver reliability of Doppler measurements, a 10% subset of study participants was randomly selected for repeated measurements. The intra-class correlation coefficient (ICC) was calculated to evaluate the reproducibility of Doppler indices, including the S/D ratio, RI, and PI. The ICC values demonstrated high intra-observer agreement, with ICC = 0.89 (95% CI: 0.84–0.93) for the S/D ratio, ICC = 0.91 (95% CI: 0.87–0.95) for RI, and ICC = 0.88 (95% CI: 0.82–0.92) for PI. These findings indicate excellent reliability and consistency of the Doppler measurements performed in this study [17].

### 2.4. Statistical Analysis

All statistical analyses were performed using the Jamovi, version 2.3.28 an open statistical software, to analyze the data. The variables were investigated using visual (histogram and probability plots) and analytic methods (Kolmogrov–Simirnov/Shapiro–Wilk’s test) to determine whether or not they are normally distributed. The Levene test was used to assess the homogeneity of the variance. For the non-normally distributed numerical data, descriptive analyses were presented using medians and quartiles (q1–q3). The Mann–Whitney U tests were conducted to compare these parameters among the groups. The capacities of various parameters that can be used to predict miscarriage, were analyzed using ROC (Receiver Operating Characteristics) curve analysis. When a significant cut-off value was observed, the sensitivity, specificity, and AUC (Area Under Curve) value were presented. A *p*-value of less than 0.05 was considered to show a statistically significant result.

## 3. Results

In the comparison of demographic and clinical characteristics between patients with abortus imminens with and without subchorionic hematoma, no statistically significant differences were observed in age, body mass index (BMI), gravida, parity, or gestational age (*p* > 0.05 for all) (Table 1). Similarly, Demographics and Clinical Characteristics were compared between pregnant women who had abortions and those whose pregnancies continued to the second trimester. No significant difference was observed between the variables (*p* > 0.05 for all) (Table 2).

Table 3 evaluates CL Doppler measurements of pregnant women who had subchorionic hematoma and had abortions in the first trimester. Median S/D ratio in the group with subchorionic hematoma was 3.07 (2.82–3.92), compared to 2.13 (1.92–2.82) in the group without. Similarly, PI was 1.11 (0.87–1.32) versus 0.80 (0.68–1.08), and RI was 0.68 (0.56–0.78) versus 0.53 (0.43–0.67) in the respective groups (*p* < 0.001 for all comparisons). When comparing patients who experienced first-trimester abortion with those whose pregnancies progressed into the second trimester, similar trends in CL Doppler parameters were observed. The median S/D ratio, PI, and RI were significantly higher in the first-trimester abortion group (3.64 [2.92–4.23], 1.28 [1.10–1.52], and 0.75 [0.65–0.92], respectively) compared to the group continuing into the second trimester (2.21 [1.98–2.93], 0.82 [0.73–0.98], and 0.54 [0.47–0.64], respectively; *p* < 0.001 for all) (Table 3).

Table 4 evaluates the relationship between corpus luteum (CL) Doppler parameters and pregnancy outcomes based on the presence or absence of subchorionic hematoma. Among pregnant women with subchorionic hematoma, those who experienced first-trimester abortion had significantly higher CL Doppler values compared to those whose pregnancies progressed into the second trimester. The median S/D ratio in the abortion group was 3.98 (3.70–4.28), compared to 2.97 (2.61–3.20) in the continuing pregnancy group (*p* < 0.001). Similarly, PI was higher in the abortion group (1.32 [1.13–1.52]) versus the continuing group (0.98 [0.82–1.23], *p* = 0.001), as was RI (0.75 [0.67–0.86] vs. 0.60 [0.52–0.71], *p* = 0.001). Among women without subchorionic hematoma, a similar pattern was observed. Those who aborted in the first trimester exhibited significantly higher CL.

Doppler parameters compared to those with ongoing pregnancies into the second trimester. The S/D ratio was 3.01 (2.55–4.12) in the abortion group versus 2.12 (1.92–2.36) in the continuing pregnancy group (*p* < 0.001). Additionally, PI was 1.26 (0.94–1.48) in the abortion group compared to 0.76 (0.67–0.87) in the continuing group (*p* < 0.001), while RI was 0.74 (0.47–0.98) versus 0.53 (0.42–0.61), respectively (*p* < 0.001). These results highlight the significant association between elevated CL Doppler parameters and adverse pregnancy outcomes, irrespective of the presence of subchorionic hematoma. ROC analysis of CL Doppler parameters demonstrated significant predictive value for subchorionic hematoma and first-trimester abortion. For predicting subchorionic hematoma, the S/D ratio cut-off value of >2.27 yielded a sensitivity of 90% and specificity of 62% (AUC = 0.774; 95% CI, 0.705–0.833; *p* < 0.001). For PI and RI, cut-off values of >0.76 and >0.53 resulted in sensitivities of 93% and 80% and specificities of 45% and 53%, respectively (*p* < 0.001 for all, Table 5). For first-trimester abortion, the optimal S/D ratio cut-off value of >2.87 demonstrated a sensitivity of 77% and specificity of 75% (AUC = 0.767; 95% CI, 0.698–0.826; *p* < 0.001). Similarly, PI > 0.93 and RI > 0.62 had sensitivities of 86% and 77% and specificities of 74% and 75%, respectively (*p* < 0.001 for all, Table 5).

**Figure 1:**
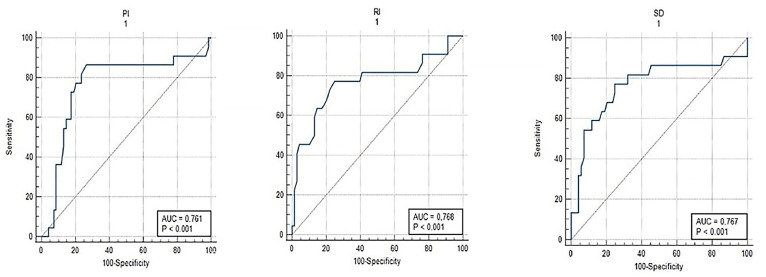
ROC analysis of CL Doppler parameters in pregnant women who have abortion in the first trimester.

**Figure 2:**
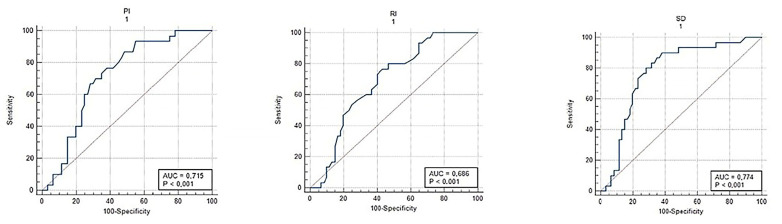
ROC analysis of corpus luteum Doppler parameters for predicting subchorionic hematoma.

**Figure 3:**
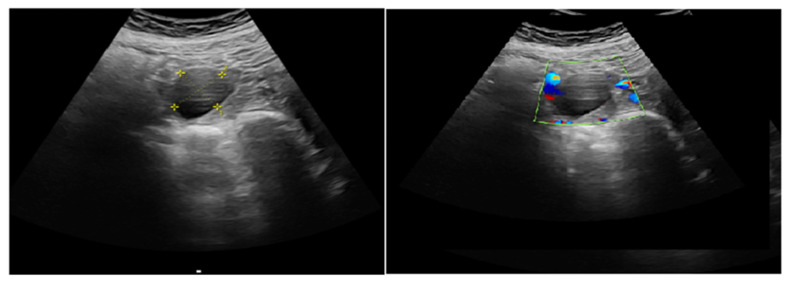
Corpus luteum ultrasound image and color Doppler image.

**Figure 4:**
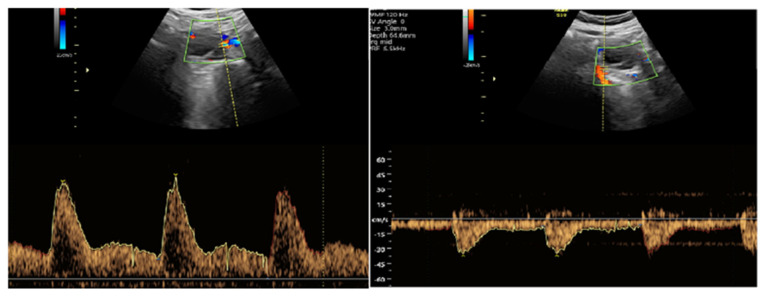
Corpus luteum power Doppler measurements.

Figure 1 illustrates the ROC analysis of corpus luteum Doppler data, indicating a substantial predictive capacity for subchorionic hematoma during the first trimester. Figure 2 illustrates the ROC analysis of corpus luteum Doppler data, indicating a substantial predictive capacity for subchorionic hematoma. Figure 3 and Figure 4 show ultrasound images and Doppler images of CL.

## 4. Discussion

In this study, we examined patients diagnosed with threatening abortion, distinguishing between those with and without subchorionic hemorrhage, by utilizing CL Doppler to assess and predict outcome. Our findings indicate that CL Doppler measurements exhibited a significant difference in patients with subchorionic hemorrhage. We also discovered that CL Doppler measurements were markedly different in individuals diagnosed with imminent abortion whose pregnancies did not progress to the second trimester, indicating its potential in prognostic prediction. Transvaginal ultrasonography is a readily available, economical, and quick technique that necessitates no further intervention for the patient, allowing for the assessment of CL and Doppler results to derive prognostic information. We believe that this can help deal with threatened miscarriage, which is one of the most common reasons for hospitalization in the first trimester and causes anxiety for mothers. To our knowledge, this is the first study to investigate pregnancy outcomes with CL Doppler in pregnancies with subchorionic hemorrhage.

Early spontaneous abortions are significant social occurrences for both the family and society. The incidence is estimated to be approximately 10–15% in patients with a confirmed clinical diagnosis of pregnancy and 30% in those with an unconfirmed clinical diagnosis [18]. CL deficiency is recognized as a significant contributor to early spontaneous abortions [19]. Despite numerous investigations into the cause, insufficient knowledge exists regarding the prevention or etiology of early abortions. Data exhibiting low specificity and sensitivity in prognostic prediction include vaginal bleeding, pelvic pain, and diminished levels of human chorionic gonadotropin (hCG) or progesterone [20].

Research indicates that the presence of subchorionic hemorrhage elevates the incidence of spontaneous abortion in individuals experiencing threatening miscarriage and correlates with adverse pregnancy outcomes. Şükür et al. showed that intrauterine hematoma is a significant prognostic indicator for the continuation of pregnancy in individuals experiencing threatened miscarriage [21]. Furthermore, Ball et al. discovered that individuals with subchorionic hematomas had an elevated incidence of preterm delivery, while Nagy et al. similarly identified that these patients experienced heightened rates of intrauterine growth restriction and preterm labor [22,23]. The subchorionic hemorrhage area is a significant risk factor for adverse pregnancy outcomes and spontaneous abortion; thus, results that aid in prognostic assessment may enhance pregnancy care. Our findings show that CL Doppler S/D, PI, and RI values are significantly higher in both patients with subchorionic hemorrhage areas and in patients with subchorionic hemorrhage who aborted. These data indicate that CL Doppler observations associated with subchorionic bleeding may predict adverse pregnancy outcomes.

The use of CL Doppler in pregnancy prediction and the identification of luteal insufficiency has been extensively studied. Alcazar et al. conducted a comparative research study between normal pregnancies and patients with missed or threatening abortions. The RI of CL dopplers exhibited a substantial disparity between patients with missed abortions and those with normal pregnancies; however, the difference between patients with threatening abortions and normal pregnancies was not statistically significant [24]. Gutiérre et al. also discovered in their investigation that the Doppler assessment of the corpus luteum was indistinguishable in pregnant women who experienced an abortion [9]. Numerous Doppler studies of uteroplacental circulation during the first trimester have not successfully predicted miscarriage [25,26]. However, there have been studies showing that pathological uterine and intraplacental Doppler findings are more common in miscarriages [27]. The uterine artery Doppler has been extensively assessed regarding its correlation with pregnancy outcomes. A study indicated that an elevated uterine vascular resistance index might forecast recurrent pregnancy loss [28]; nevertheless, another study found no distinction in uterine artery Doppler between pathological and normal pregnancies [12]. In their study, Mansour et al. noted that endometrial and subendometrial ischemia might develop in recurrent pregnancy losses, and this can be monitored with uterine artery Doppler [29]. Recent investigations have provided compelling evidence that the usage of low-dose aspirin has risen and that it influences uterine artery blood flow [30]. A study assessing the uterine and spiral arteries indicated that recurrent pregnancy loss correlates with the blood flow in these vessels, suggesting that the parameters of the spiral arteries may be more dependable [31]. The literature also states that there is a correlation between the presence of a subchorionic bleeding area and abortion, but there is no difference between the CL Dopplers of these patients [32]. In addition to all these studies, there are also studies that show that the RI and PI values of the CL Doppler are high in patients with threatened abortion and miscarriage [33,34]. Han and colleagues, in their investigation, revealed that CL RI values can serve as a significant indicator for predicting pregnancy outcomes [35]. As an alternative to these investigations, our research examined the role of CL Doppler in prognosis between patients with and without subchorionic hemorrhage. The disparity in RI and PI values between the two groups was statistically significant, as was the distinction between pregnant women who underwent an abortion in the first trimester and those whose pregnancies progressed into the second trimester.

CL Doppler investigations have been utilized to illustrate luteal phase deficiencies. Another study indicated that employing a grading system based on the vascularization signal surrounding the corpus luteum could predict missed or incomplete abortions more efficiently than assessing blood progesterone levels [36]. Numerous studies indicate that in patients with luteal phase deficiencies, CL RI was markedly elevated compared to those without such problems and had a negative correlation with serum progesterone levels [34,37]. Biochemical markers have been utilized in numerous studies to forecast prognosis and pregnancy problems in cases of threatened miscarriage [38]. Serum beta-HCG, estradiol, PAPP-A, inhibin, CA 125, progesterone are some of the markers examined [39]. Ku and colleagues in their study of 929 patients indicated that low serum progesterone levels may correlate with threatening miscarriage and abortions prior to the 16th week of gestation [40]. Given that the corpus luteum (CL) is the primary source of progesterone until the complete transition to placental function, these findings corroborate our results and imply that the observed increase in resistance in CL Doppler may indirectly signify a reduction in progesterone levels. 

This study had some limitations. First, the sample size was relatively limited. Second, we could not measure progesterone levels simultaneously with CL Doppler measurements and, therefore, could not examine the correlation between Doppler values and progesterone levels. Future studies may overcome this limitation by looking at blood progesterone levels simultaneously with Doppler measurements. This study’s strength is in the novel evaluation of CL Doppler measures in cases of imminent abortion accompanied by subchorionic hematoma, a first in the literature to our knowledge.

## 5. Conclusions

In conclusion, managing patients with imminent miscarriage necessitates a personalized initial risk assessment, careful selection for hospitalization, and an evaluation of prognosis to guide recommendations for further therapy. The significance escalates when we acknowledge that this patient demographic is among the most prevalent causes of hospital admissions during pregnancy. The findings of this study provide valuable insights into the prognostic role of CL Doppler measurements in threatened abortion; however, the generalizability of these results should be considered within certain limitations. This study was conducted in a single-center setting with a relatively homogeneous patient population, which may limit its applicability to broader populations with diverse demographic and clinical characteristics. Additionally, while strict inclusion and exclusion criteria helped minimize confounding factors, they may have also resulted in a study cohort that does not fully represent the variability seen in clinical practice. The use of a standardized Doppler measurement protocol by a single experienced sonographer enhances the reliability of the results, but further multicenter studies involving larger and more diverse populations are necessary to confirm the external validity of these findings. Future research should also explore whether variations in CL Doppler parameters across different populations (e.g., different ethnic groups, gestational ages, or underlying risk factors) affect pregnancy outcomes similarly.

## Figures and Tables

**Table 1:** Demographic characteristics of the study participant.

Variables	Abortus Imminens Without Subchorionic Bleeding Area *n* = 120	Abortus Imminens with Subchorionic Bleeding Area *n* = 60	*p*
Age median (q_1_–q_3_)	27 (24–29)	26 (23–31)	0.803 ^Φ^
BMI (kg/m^2^) median (q1–q3)	27.8 (23.5–31.3)	27.1 (24.5–30.0)	0.468 ^Φ^
Gravida median (q_1_–q_3_)	2 (1–3)	2 (1–3)	0.165 ^Φ^
Parity median (q_1_–q_3_)	0 (0–1)	1 (0–1)	0.388 ^Φ^
Gestational week (according to ultrasound) median (q1–q3)	8 (6–10)	8 (7–9)	0.731 ^Φ^
Ectopic pregnancy history median (q1–q3)	0 (0–0)	0 (0–0)	0.086 ^Φ^
TSH median (q1–q3)	1.40 (1.10–2.78)	1.59 (1.16–1.96)	0.818 ^Φ^

^Φ^ Mann–Whitney U test. TSH: thyroid-stimulating hormone, BMI: body mass index. Data are expressed as median and quartiles (q1–q3). A *p* value of <0.05 indicates a significant difference.

**Table 2:** Comparison of maternal demographics and clinical characteristics between abortus patients and those continuing pregnancy into the second trimester.

Variables	Pregnant Women Who Have Abortıon in the First Trımester *n* = 44	Those Who Contınue Theır Pregnancy in the Second Trımester *n* = 136	*p*
Age median (q_1_–q_3_)	26 (24–30)	27 (24–30)	0.989 ^Φ^
BMI (kg/m^2^) median (q1–q3)	26.8 (23.3–28.8)	28.0 (24.2–31.2)	0.076 ^Φ^
Gravida median (q_1_–q_3_)	2 (1–4)	2 (1–3)	0.465 ^Φ^
Parity median (q_1_–q_3_)	1 (0–1)	0 (0–1)	0.087 ^Φ^
Gestational week (according to ultrasound) median (q1–q3)	7 (6–9)	9 (7–10)	0.061 ^Φ^
Ectopic pregnancy history median (q1–q3)	0 (0–0)	0 (0–0)	0.158 ^Φ^
TSH median (q1–q3)	1.50 (1.23–1.70)	1.48 (1.08–2.78)	0.799 ^Φ^

^Φ^ Mann–Whitney U test. TSH: thyroid-stimulating hormone, BMI: body mass index. Data are expressed as median and quartiles (q1–q3). A *p* value of <0.05 indicates a significant difference.

**Table 3:** Comparison of corpus luteum Doppler parameters in pregnancy outcomes based on the presence of subchorionic hematoma and first trimester abortion risk.

Variables	Pregnant Women Who Have an Abortıon in the First Trımester *n* = 44	Those Who Contınue Their Pregnancy in the Second Trımester *n* = 136	*p*
CL S/D ratio	3.64 (2.92–4.23)	2.21 (1.98–2.93)	**<0.001 ^Φ^**
CL PI	1.28 (1.10–1.52)	0.82 (0.73–0.98)	**<0.001 ^Φ^**
CL RI	0.75 (0.65–0.92)	0.54 (0.47–0.64)	**<0.001 ^Φ^**
**Variables**	**Abortus imminens without subchorionic bleeding area** ***n* = 120**	**Abortus imminens with subchorionic bleeding area** ***n* = 60**	***p***
CL S/D ratio	2.13 (1.92–2.82)	3.07 (2.82–3.92)	**<0.001 ^Φ^**
CL PI	0.80 (0.68–1.08)	1.11 (0.87–1.32)	**<0.001 ^Φ^**
CL RI	0.53 (0.43–0.67)	0.68 (0.56–0.78)	**<0.001 ^Φ^**

^Φ^ Mann–Whitney U test. CL: corpus luteum, S/D ratio: systolic/diastolic (S/D) ratio, RI: Resistivity Index, PI: ulsatility Index (PI). Statistically significant *p*-values are in bold.

**Table 4:** Relationship between corpus luteum Doppler parameters and pregnancy outcomes based on the presence or absence of subchorionic hematoma.

Variables	Pregnant Women with Subchorionic Bleeding Areas and Who Abort in the First Trimester *n* = 18	Continuing Pregnancy into the 2nd Trimester with a Subchorionic Bleeding Area *n* = 42	*p*
CL S/D ratio	3.98 (3.70–4.28)	2.97 (2.61–3.20)	**<0.001 ^Φ^**
CL PI	1.32 (1.13–1.52)	0.98 (0.82–1.23)	**0.001 ^Φ^**
CL RI	0.75 (0.67–0.86)	0.60 (0.52–0.71)	**0.001 ^Φ^**
**Variables**	**Pregnant women who have no subchorionic bleeding area and who abort in the first trimester** ***n* = 26**	**Continuing pregnancy into the 2nd trimester without a subchorionic bleeding area** ***n* = 94**	***p***
CL S/D ratio	3.01 (2.55–4.12)	2.12 (1.92–2.36)	**<0.001 ^Φ^**
CL PI	1.26 (0.94–1.48)	0.76 (0.67–0.87)	**<0.001 ^Φ^**
CL RI	0.74 (0.47–0.98)	0.53 (0.42–0.61)	**<0.001 ^Φ^**

^Φ^ Mann–Whitney U test. CL: corpus luteum, S/D ratio: systolic/diastolic (S/D) ratio, RI: Resistivity Index, PI: ulsatility Index (PI). Statistically significant *p*-values are in bold.

**Table 5:** ROC Analysis of Corpus Luteum Doppler Parameters for Predicting Subchorionic Hematoma and Abortus.

Variables	AUC	95% CI	Cut-Off	*p*	Sensitivite	Spesifite
Abortus imminens with subchorionic bleeding area						
S/D	0.774	0.705–0.833	>2.27	<0.001	90	62
PI	0.715	0.643–0.780	>0.76	<0.001	93	45
RI	0.686	0.613–0.753	>0.53	<0.001	80	53
Pregnant women who have abortıon ın the First trımester						
S/D	0.767	0.698–0.826	>2.87	<0.001	77	75
PI	0.761	0.692–0.822	>0.93	<0.001	86	74
RI	0.768	0.699–0.827	>0.62	<0.001	77	75

CL: corpus luteum, S/D ratio: systolic/diastolic (S/D) ratio, RI: Resistivity Index, PI: Pulsatility Index (PI), AUC: Area Under the Curve.

## Data Availability

The data supporting this study are available from the corresponding authors upon reasonable request.

## Abbreviations

AUC	Area Under Curve
BMI	Body Mass Index
CL	Corpus Luteum
CRL	Crown-Rump Length
hCG	Human Chorionic Gonadotropin
PI	Pulsatility Index
RI	Resistance Index
ROC	Receiver Operating Characteristics
S/D	Systole/Diastole Ratio
